# The macrolide drug erythromycin does not protect the hERG channel from inhibition by thioridazine and terfenadine

**DOI:** 10.14814/phy2.14385

**Published:** 2020-03-08

**Authors:** Aziza El Harchi, Andrew S. Butler, Yihong Zhang, Christopher E. Dempsey, Jules C. Hancox

**Affiliations:** ^1^ School of Physiology and Pharmacology and Neuroscience Biomedical Sciences Building The University of Bristol University Walk Bristol UK; ^2^ School of Biochemistry Biomedical Sciences Building The University of Bristol University Walk Bristol UK

**Keywords:** allosteric interaction, BeKm‐1, erythromycin, hERG, long QT, potassium channel

## Abstract

The macrolide antibiotic erythromycin has been associated with QT interval prolongation and inhibition of the hERG‐encoded channels responsible for the rapid delayed rectifier K^+^ current I(_Kr_). It has been suggested that *low* concentrations of erythromycin may have a protective effect against hERG block and associated drug‐induced arrhythmia by reducing the affinity of the pore‐binding site for high potency hERG inhibitors. This study aimed to explore further the notion of a potentially protective effect of erythromycin. Whole‐cell patch‐clamp experiments were performed in which hERG‐expressing mammalian (Human Embryonic Kidney; HEK) cells were preincubated with low to moderate concentrations of erythromycin (3 or 30 µM) prior to whole‐cell patch clamp recordings of hERG current (I_hERG_) at 37°C. In contrast to a previous report, exposure to low concentrations of erythromycin did not reduce pharmacological sensitivity of hERG to the antipsychotic thioridazine and antihistamine terfenadine. The IC_50_ value for I_hERG_ tail inhibition by terfenadine was decreased by ~32‐fold in the presence of 3 µM erythromycin (*p* < .05 vs. no preincubation). Sensitivity to thioridazine remained unchanged (*p* > .05 vs. no preincubation). The effects of low concentrations of erythromycin were investigated for a series of pore blocking drugs, and the results obtained were consistent with additive and/or synergistic effects. Experiments with the externally acting blocker BeKm‐1 on WT hERG and a pore mutant (F656V) were used to explore the location of the binding site for erythromycin. Our data are inconsistent with the use of erythromycin for the management of drug‐induced QT prolongation.

## Graphical abstract

In contrast to an earlier study that suggested a protective effect of the macrolide antibiotic erythromycin against pharmacological block of recombinant hERG channels overexpressed in Human Embryonic Kidney (HEK) cells, the present report showed that pretreatment with low concentrations of erythromycin did not reduce hERG channel sensitivity to inhibitors. In the presence of erythromycin, hERG displayed increased sensitivity to pharmacological inhibition by three drugs: the antihistamine terfenadine, the antimalarial chloroquine and antiarrythmic dofetilide. hERG block by antipsychotic thioridazine and antifungal ketoconazole was unaffected. This complex pharmacological profile of erythromycin actions may be mediated through two distinct binding sites on the hERG channel.

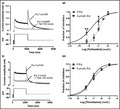

## 1 INTRODUCTION

For a number of species in which the cardiac ventricular action potential (AP) exhibits a high plateau phase (including guinea‐pig, rabbit, dog and—importantly—human), the rapid delayed rectifier K^+^ current (I_Kr_) contributes significantly to ventricular AP repolarization and to setting the duration of the QT interval of the ECG (Gima & Rudy, 2002; Sanguinetti & Jurkiewicz, 1991; Tamargo, Caballero, Gomez, Valenzuela, & Delpon, 2004; Virag et al., 2001). The pore‐forming (α) subunit of I_Kr_ is encoded by hERG (*human Ether‐à‐go‐go Related Gene*; alternative nomenclature *KCNH2*) and functional channels are comprised of hERG subunit tetramers (Sanguinetti, Jiang, Curran, & Keating, 1995). Unique structural features, including the presence of specific aromatic amino acid residues (tyrosine at 652 & phenylalanine at 656; Y652 and F656) in the S6 helices of the channel, are believed to contribute to hERG’s sensitivity to pharmacological blockade (Hancox, McPate, El Harchi, & Zhang, 2008; Sanguinetti & Tristani‐Firouzi, 2006; Vandenberg, Walker, & Campbell, 2001). Structurally diverse drugs in clinical use produce hERG blockade as an unwanted side effect. This carries a risk of prolongation of the QT interval of the electrocardiogram which, in turn, is associated with a risk of the potentially fatal arrhythmia Torsade de Pointes (TdP) (Hancox et al., 2008; Vandenberg et al., 2001). The treatment of drug‐induced long QT syndrome (diLQTS) and TdP is largely supportive until levels of the drug in question have fallen along with the QT interval (Yap & Camm, 2003). A drug that could selectively reverse hERG channel blockade would therefore be an attractive adjunct or replacement treatment as well as being able to provide novel insight into how hERG blockade might be avoided in drug development. In recent years a number of hERG channel agonists (activators) have been identified. In principle, such drugs could produce “functional” antagonism of hERG blocking drugs (Meng, Shi, Li, Du, & Xu, 2013), not necessarily through impairing drug‐binding per se, but by increasing current through unblocked channels.

It has been suggested that erythromycin, a macrolide antibiotic drug, which in high concentrations can cause QT interval prolongation (Crumb, 2014), may at *low* concentrations have a protective effect against hERG block by reducing the affinity of the pore‐binding site for high potency hERG inhibitors with no apparent change to hERG kinetics (Crumb, 2014). The effects of two potent hERG inhibitors (the antipsychotic thioridazine and the withdrawn antihistamine terfenadine) on I_hERG_ were found to be attenuated by pretreatment of a hERG‐expressing mammalian cell line with erythromycin (at erythromycin concentrations producing 10% or less block of I_hERG_) (Crumb, 2014). The results of that study were interpreted to raise the possibility of antagonistic allosteric interactions between drug‐binding sites on the external and internal face of the hERG channel (Crumb, 2014) and it is possible for hERG‐blocking compounds to have relatively weak interactions with residues of the canonical drug‐binding site (Duncan et al., 2006; Mitcheson, 2003). However, at present there is only limited evidence for the putative protective effect of erythromycin and its associated site of action. To inform conceptual development of treatment approaches to diLQTS, we aimed in this study to: (a) characterize erythromycin's potential interactions with a selection of pore and nonpore hERG inhibitors and (b) provide further information as to where on the channel erythromycin potentially interacts to modulate hERG pore blocker action.

## 2 MATERIAL AND METHODS

### 2.1 Mutagenesis

The F656V hERG mutation (Lees‐Miller, Duan, Teng, & Duff, 2000) was generated using the QuickChange® site‐directed mutagenesis kit (Stratagene). In brief, a pair of complementary oligonucleotide primers containing the mutation (forward primer sequence 5′GTATGCTAGCATCGTCGGCAACGTGTCG3′ and reverse primer sequence 5′ CGACACGTTGCCGACGATGCTAGCATAC3′, synthesized by Sigma‐Genosys) was used in a PCR reaction (95°C for 1 min, 60°C for 1 min, 68°C for 16 min for 18 cycles) with hERG in a modified pcDNA3.0 vector as a DNA template. A DpnI digest of the PCR mix was then performed for 1 hr at 37°C. Competent DH5*α Escherichia coli* (Invitrogen) were transformed using standard procedures. The mutation was confirmed by sequencing of the entire open reading frame (Eurofins MWG Operon).

### 2.2 Maintenance of HEK cells and cell transfection

Human Embryonic Kidney (HEK‐293) cells stably expressing WT hERG were kindly donated by Prof Craig January. HEK 293 cells used for transient transfection were obtained from ECACC (catalog number 85120602). Cells were passaged using a nonenzymatic agent (Enzyme Free, Chemicon International^®^) and maintained as previously described (McPate, Duncan, Milnes, Witchel, & Hancox, 2005; Milnes, Crociani, Arcangeli, Hancox, & Witchel, 2003; Ridley, Dooley, Milnes, Witchel, & Hancox, 2004). For transient transfection experiments, 24 hr after plating cells out, cells were transiently transfected with 0.5 µg of the F656V hERG construct using Lipofectamine^TM^ LTX (Invitrogen) according to the manufacturer's instructions. Expression plasmid encoding CD8 was also added as a transfection marker (El Harchi, Zhang, Hussein, Dempsey, & Hancox, 2012). Cells were plated onto small sterilized glass coverslips 6 hr after transfection and recordings were made after at least 24 hr incubation at 37°C. Successfully transfected cells were identified using Dynabeads^®^ (Invitrogen). All experimental data for the F656V hERG mutant channel were obtained from cells from a minimum of two transfections.

### 2.3 Electrophysiological recordings

For whole‐cell patch‐clamp recording cells were continuously superfused at physiological temperature (37°C) with an external solution containing (in mM): 140 NaCl, 4 KCl, 2.5 CaCl_2_, 1 MgCl_2_, 10 Glucose and 5 HEPES (titrated to pH 7.45 with NaOH). Patch‐pipettes (Corning 7052 glass, AM Systems) were pulled and heat‐polished (Narishige MF83) to 2.5–4 MΩ; pipette dialysate contained (in mM): 130 KCl, 1 MgCl_2_, 5 EGTA, 5 MgATP, 10 HEPES (titrated to pH 7.2 using KOH). Recordings of hERG current (I_hERG_) and were made using an Axopatch 200 amplifier (Axon Instruments) and a CV201 head‐stage. Between 70%–80% of pipette series resistance was compensated. Voltage‐clamp commands were generated using “WinWCP” (John Dempster, Strathclyde University).

### 2.4 Drug selection and preparation

Erythromcyin's actions were investigated for a range of drugs selected on the basis of their known arrythmogenic potential, potency of block, interaction site within the pore of the hERG channel and/or state‐dependent kinetics of block. The withdrawn antihistamine terfenadine and antipsychotic thioridazine (nM IC_50_) (Crumb, 2000; Milnes, Witchel, Leaney, Leishman, & Hancox, 2006) were selected to test the reported effect of low concentrations of erythromycin on I_hERG_ block by these drugs under our conditions (Crumb, 2014). Dofetilide (high affinity [nM IC_50_]) (Milnes, Witchel, Leaney, Leishman, & Hancox, 2010) is an archetypal selective (“methanesulfonanilide”) hERG blocker, with some risk of TdP (Hancox et al., 2008; Yap & Camm, 2003). Chloroquine (low μM IC_50_) is an antimalarial agent that exhibits a fast‐open block for the hERG channel (Sánchez‐Chapula, Navarro‐Polanco, Culberson, Chen, & Sanguinetti, 2002) and can prolong the QT interval (Bustos, Gay, Diquet, Thomare, & Warot, 1994; Khobragade et al., 2013). The antifungal ketoconazole (low μM IC_50_) is an imidazole relative of clotrimazole (previously reported to produce additive or slightly synergistic block of I_hERG_ in combination with erythromycin (Crumb, 2014), but unlike clotrimazole is known to bind within the pore (Ridley et al., 2006). Disopyramide (low μM IC_50_) is a class Ia antiarrythmic agent inhibitor that binds low in the hERG channel's inner cavity, which renders the inhibitory effects of disopyramide poorly dependent on inactivation (El Harchi et al., 2012; McPate et al., 2005). The peptide toxin BeKm‐1 exhibits preferential closed channel block (Milnes, Dempsey, et al., 2003). This toxin was selected because it interacts with the S5‐pore linker/outer vestibule of the channel from the external surface (Tseng et al., 2007).

Erythromycin was dissolved in ethanol (Sigma) to produce a stock solution of 50 mM. Chloroquine and disopyramide‐diphosphate (Sigma‐Aldrich) were dissolved in deionized water (Milli‐Q, Millipore) at a stock concentration of 50 mM. Terfenadine, ketoconazole and thioridazine (Sigma‐Aldrich) were dissolved in methanol to produce stock solutions of 10 mM. All stock solutions were diluted to produce stock solutions ranging down to 1 mM with at least 1:1000‐fold final dilution with Tyrode's solution to achieve concentrations stated in the Results. At such dilutions, the stock solution vehicles used have been shown not to significantly affect I_hERG_ amplitude (Himmel, 2007; Tie et al., 2000). In house Fmoc‐synthesized BeKm‐1 (Milnes, Dempsey, et al., 2003) was dissolved in sterile 10 mM Tris–HCl, 1 mM EDTA, pH 7.6 to produce stock solutions of 25 μM. Stock solutions were aliquoted into glass vials and stored at −20°C before use (Milnes, Dempsey, et al., 2003). External solutions were applied using a home‐built, warmed, and rapid solution exchange device (Levi, Hancox, Howarth, Croker, & Vinnicombe, 1996).

### 2.5 Electrophysiology data analysis

Concentration–response relations were fitted with a standard Hill equation to obtain half‐maximal inhibitory concentration (IC_50_) and Hill‐coefficient (n_H_) values (GraphPad Prism v.7) of the form:

(1) $$\mathrm{Fractional}\;\mathrm{block}=1/\left(1+\left(IC_{50}/\left[\mathrm{DRUG}\right]\right){\;}^{{}^{\mathrm{nH}}}\right)$$

Drug combination index (DCI) values were calculated to measure and quantify the effects on I_hERG_ produced by drug combination, using the following equation (Chou, 2006):

(2) $$\text{DCI}=A_{1}/A_{\left(x\right)1}+B_{1}/B_{\left(x\right)1}$$

where the denominators A_(*x*)1_ and B_(*x*)1_ stand for the concentrations of test substances A and B, with each inhibiting hERG peak tail currents by *x*%, and the numerators are concentrations A_1_ and B_1_ which, in combination, also inhibit hERG peak tail currents by *x*%. The A_(*x*)1_ and B_(*x*)1_ values were derived from Equation 1. A Combination Index value equal to 1 indicates additivity. A value greater or smaller than 1 indicates, respectively, antagonism or synergism (Chou, 2006).

Time courses of I_hERG_ inhibition were fitted using either a single (Equation 3) exponential decay fit of the forms:

(3) $$Y=\left(Y_{{}^{0}}-\mathrm{Plateau}\right)*\exp\left(-K*X\right)+\mathrm{Plateau}.$$

Mean values in the text are presented either as mean ± *SEM* or (for IC_50_ and n_H_ values) as mean ± 95% confidence intervals (CI) from *n* = 4 to 10 cells per experiment. Statistical analysis was performed using unpaired *t* tests or one‐way and two‐way analysis of variance as appropriate (Sigmaplot v13) with Bonferroni post hoc test. Tests used are specified in the relevant figure legends. P values of less than 0.05 were taken as statistically significant.

## 3 RESULTS

### 3.1 hERG sensitivity to erythromycin, terfenadine, and thioridazine

**Figure 1:**
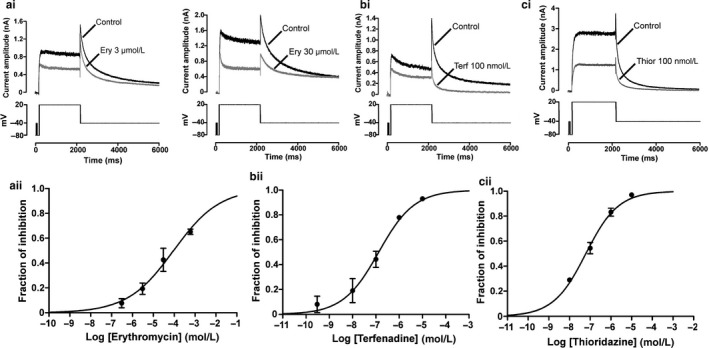
Sensitivity of I_hERG_ to erythromycin (a), terfenadine (b) and thioridazine (c). Top panels show representative traces for I_hERG_ recorded at 37ºC before and during exposure to 3 or 30 µM erythromycin (Ery) (ai), 100 nM terfenadine (Terf) (bi) or 100 nM thioridazine (Thior) (ci). Currents were elicited using the protocol shown in the lower panels. Bottom panels show the isochronal concentration–response relationships obtained for erythromycin (aii) (IC_50_ 94 µM (CI 38.4–230.1 µM); *n* = 5 to 8 to cells per concentration), terfenadine (bii) (IC_50_ 128.5 nM (CI 68.0–243.3 nM); *n* = 4 to 6 to cells per concentration) and thioridazine (cii) (IC_50_ 62 nM (CI 52.7–73.1 nM); *n* = 5 to 7 cells per concentration). Respective n_H_ values yielded from fit were of 0.40 (CI 0.25–0.54), 0.57 (CI 0.36–0.77), 0.55 (CI 0.50–0.61) for erythromycin, for terfenadine and for thioridazine

Figure 1 shows the concentration dependence of I_hERG_ inhibition by erythromycin, terfenadine and thioridazine under the recording conditions of this study, with representative traces of effects of single drug concentrations shown in Figure 1ai–ci and concentration–response relations shown in Figure 1aii–cii. I_hERG_ traces were elicited by the voltage protocol shown in the lower panels of Figure 1ai–ci (a standard command protocol used in previous studies of I_hERG_ pharmacology from our laboratory (e.g., El Harchi et al., 2012; Milnes et al., 2010; Zhang, Colenso, Sessions, Dempsey, & Hancox, 2011)). I_hERG_ “tail” magnitude was measured relative to instantaneous current at −40 mV elicited by the brief (50 ms) depolarizing step that preceded the voltage command to +20 mV in the absence and presence of the drug. Consistent with a previous report from our laboratory (Duncan et al., 2006), 3 µM and 30 µM erythromycin reduced I_hERG_ tail, respectively, by 22.5 ± 3.5% (*n* = 8 cells) and 42.6 ± 9.3% (*n* = 6 cells) within 6‐min of drug superfusion. A range of erythromycin concentrations between 0.3 and 600 µM was then tested and a concentration response relation constructed. The half maximal inhibitory concentration (IC_50_) derived from a standard Hill equation was 94 μM (CI 38.4 μM to 230.1 μM; *n* = 5 to 8 cells per concentration) with a Hill coefficient (n_H_) of 0.40 (CI 0.25–0.54; *cf* [Stanat, Carlton, Crumb, Agrawal, & Clarkson, 2003]). This is in fair agreement with previously published IC_50_ values of 59.3 μM (Duncan et al., 2006) and 72.2 µM (Volberg, Koci, Su, Lin, & Zhou, 2002). A fit to the concentration–response data for inhibition of I_hERG_ tail by terfenadine yielded an IC_50_ value of 128.5 nM (CI 68.0–243.3 nM; *n* = 4 to 6 cells per concentration) and an n_H_ of 0.57 (CI 0.36–0.77 nM). Finally, thioridazine was found to inhibit I_hERG_ tails with an IC_50_ of 62 nM (CI 52.7–73.1 nM; *n* = 5 to 7 cells per concentration) and an n_H_ of 0.55 (CI 0.50–0.61). We also assessed the effects of drug block on I_hERG_ time constants of deactivation. Tail currents on repolarization to −40mV were fitted using a biexponential to derive the fast τ_1_ and slow τ_2_ time constants of deactivation in control and after drug application. The estimated fast time constant τ_1_ and slow time constant τ_2_ were, respectively, 158.0 ± 20.5 ms and 1,196.1 ± 103.1 ms in control (*n* = 6 cells). At 6‐min of exposure to 30 µM erythromycin, the fast component of deactivation was slowed but the slow component remained unchanged: τ_1_ was 232.4 ± 33.6 ms (*n* = 6 cells; *p* < .05 vs. control) and τ_2_ was 1,335.1 ± 185.4 ms in erythromycin (*n* = 6; cells *p* > .05 vs. control). The proportion of deactivating current fitted by the fast component *τ_1_*_ _was ∼0.6 and ~0.4 for the slow component τ_2._ None of these values was significantly different between control and following drug application (*p* > .05 vs. in control). No change to the time constants of deactivation was observed following exposure to 100 nM terfenadine or 100 nM thioridazine (*p* > .05 and *p* > .05 vs. control; *n* = 6 and 7 cells, respectively).

### 3.2 Sensitivity to terfenadine and thioridazine of I_hERG_ recorded from erythromycin pretreated cells

**Figure 2:**
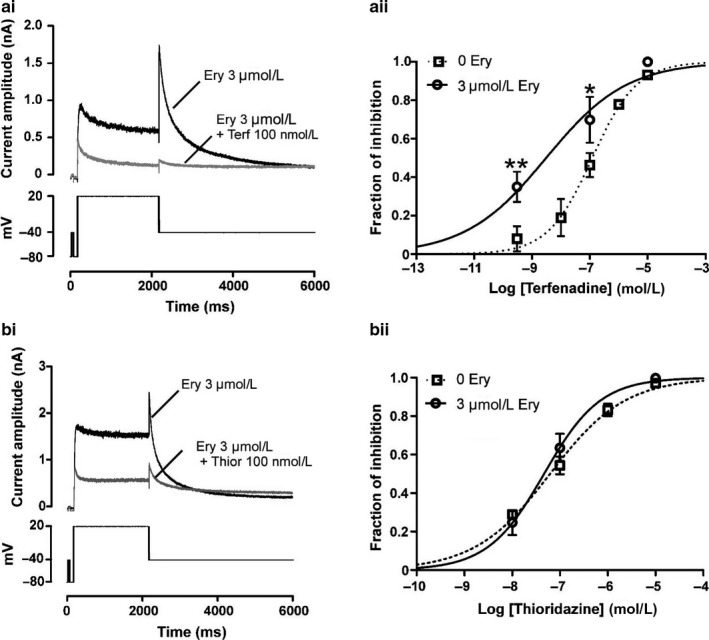
Effect of a pretreatment with 3 µM erythromycin on I_hERG_ block by terfenadine (a) and thioridazine (b). Left panels show representative current traces for effects of 100 nM terfenadine (ai) or 100 nM thioridazine (bi) on I_hERG_ after a 15‐min preincubation with 3 µM erythromycin. Currents were elicited using the protocol shown in the lower panels. Right panels show isochronal concentration–response relationships for terfenadine inhibition of I_hERG_ (aii) or thioridazine (bii) in the presence of 3 µM erythromycin. Fit to the concentration–response curves yielded an IC_50_ of 4 nM ((CI 0.63–25.6 nM); *n* = 5 cells per concentration) for terfenadine and 46.4 nM ((CI 36.3–59.3 nM); *n* = 4 to 6 cells per concentration) for thioridazine. Respective n_H_ were of 0.30 (CI 0.14–0.47) for terfenadine and of 0.73 (CI 0.58–0.90) for thioridazine. Asterisks in Aii denote statistical significance: ** at *p* < .01 and *p at < .05 (two‐way ANOVA with Bonferroni post hoc test)

Since the substantial inhibitory effect on I_hERG_ at higher concentrations (≥30 μM) would preclude the use of such erythromycin concentrations to protect against hERG channel block by other drugs, we predominantly focused our investigation on the effects of a low concentration of 3 μM erythromycin, as reported in (Crumb, 2014). Figure 2 shows the effects of a 15‐min preincubation with 3 µM erythromycin on the concentration dependence of I_hERG_ inhibition by terfenadine and thioridazine, with representative traces of effects of single drug concentrations shown in Figure 2ai–bi and concentration–response relations shown in Figure 2aii–bii. 100 nM terfenadine reduced I_hERG_ tail by 66.7 ± 11.1% (*n* = 5; *p* > .05 vs. in the absence of erythromycin). Two other terfenadine concentrations were tested in the continuous presence of 3 µM erythromycin and a concentration–response relation was constructed, yielding an IC_50_ of 4 nM (CI 0.63–25.6 nM; *n* = 5 cells per concentration) with a Hill coefficient (n_H_) of 0.3 (CI 0.14–0.47). Superimposition (as dashed line) of the concentration–response relation for inhibition of I_hERG_ tail in the absence of 3 µM erythromycin was made to allow comparison between the two conditions. These data indicate that, under the conditions of this study, exposure to a low concentration of erythromycin resulted in a ~32‐fold increase in I_hERG_ sensitivity to terfenadine as shown in Figure 2aii. In order to assess further the effects of a pretreatment with 3 µM erythromycin on hERG sensitivity to terfenadine, we also calculated the drug combination index (DCI); recognized as a gold standard measure of drug combination effects (Chou, 2006): a DCI of 0.06 was obtained which would be indicative of strong synergism (Chou, 2006).

In contrast to its effect on terfenadine, 3 µM erythromycin exposure did not affect I_hERG_ block by thioridazine. The IC_50_ for I_hERG_ tail inhibition by thioridazine in the presence of erythromycin was 46.4 nM (CI 36.3–59.3 nM; *n* = 4 to 6 cells per concentration; n_H_ = 0.73 [CI 0.58–0.90]) which does not differ from the calculated IC_50_ value for I_hERG_ inhibition in the absence of erythromycin as represented by the respective overlapping concentration–response curves in Figure 2bii (*p* > .05).

### 3.3 Effects of erythromycin on I_hERG_ inhibition by different pore blocking inhibitors

The differences in effects of preincubation with 3 µM erythromycin on I_hERG_ sensitivity to terfenadine and thioridazine suggests that results of erythromycin exposure could vary between different pore‐blocking drugs. Additional experiments were therefore conducted to assess the effects of low concentrations of erythromycin (i.e., ≤30 µM) on I_hERG_ tail block by a series of hERG pore inhibitors exhibiting varying blocking/interaction properties (for selection see also Methods). In addition to terfenadine and thioridazine, dofetilide, chloroquine, and ketoconazole were tested. Results obtained under conventional voltage clamp for all five drugs are summarized in Table 1. Effects were studied of preincubation with either 3 µM or 30 µM erythromycin on I_hERG_ sensitivity to a concentration close to the IC_50_ value for each of the different blockers selected. These experiments showed that increasing erythromycin concentration from 3 to 30 µM had limited effect on terfenadine and thioridazine block. I_hERG_ block by dofetilide was significantly increased in the presence of erythromycin. Thus, 10 nM dofetilide reduced I_hERG_ tails by 52.1 ± 3.0% (*n* = 6). In the presence of 3 µM or 30 µM erythromycin, I_hERG_ block by dofetilide was significantly increased, respectively, to 66.0 ± 4.8% (*n* = 5, *p* < .05 vs. in the absence of erythromycin) and 68.9 ± 2.8% (*n* = 5 cells, *p* < .01 vs. in the absence of erythromycin and *p* > .05 vs. in the presence of 3 µM erythromycin). Similarly, exposure to 3 µM erythromycin or 30 µM erythromycin was associated with increased hERG block by chloroquine (Table 1). Finally, sensitivity to ketoconazole of I_hERG_ tail recorded from erythromycin‐pretreated cells was similar to that of I_hERG_ recorded in the absence of the macrolide (Table 1). Given the lack of protective effect of a pretreatment with erythromycin, we also checked whether reversing the order of application could influence results (data not shown). Superfusion of 3 µM erythromycin following steady state of hERG block was associated either with additive drug effects for some pore inhibitors or no effect for others. Erythromycin at a single concentration of 3 µM failed to block currents exposed to 100 nM terfenadine or 3 µM ketoconazole. In contrast, superfusion of 3 µM erythromycin was associated with 43.4 ± 6.1% (*n* = 5 cells; *p* < .05 vs. in the absence of dofetilide) and 70.0 ± 11.7% (*n* = 5 cells; *p* < .01 vs. in the absence of thioridazine) of hERG block, respectively, in the presence of 10 nM dofetilide or 100 nM thioridazine. In the presence of 1 µM chloroquine, the percentage of I_hERG_ tail inhibition was 43.7 ± 3.0% (*n* = 5; *p* < .01 vs. in the absence of chloroquine). Overall, our data suggest that under the conditions of this study low concentrations of erythromycin had no protective effect against pharmacological blockade of hERG. For some inhibitors results were consistent with additive and/or possibly synergistic interactions that vary with the order of application and the nature of the pore inhibitor tested.

**Table 1:** Isochronal percentage of hERG block by diverse hERG pore blockers in absence or in presence of erythromycin

[Erythromycin]	0 µM	3 µM	30 µM
Terfenadine 100 nM	45.0 ± 5.2 (n = 6)	66.7 ± 11.8 (n = 5)	71.4 ± 9.7 (n = 5)
Thioridazine 100 nM	54.4 ± 4.6 (n = 6)	63.5 ± 7.3 (n = 6)	63.3 ± 4.3 (n = 5)
Dofetilide 10 nM	52.1 ± 3.0 (n = 6)	66.0 ± 4.8 (n = 5)*	68.9 ± 2.8 (n = 5)**
Chloroquine 1 µM	48.4 ± 3.9 (n = 5)	70.8 ± 5.3 (n = 5)***	77.8 ± 0.6 (n = 4)***
Ketoconazole 3 µM	47.5 ± 5.0 (n = 7)	59.5 ± 3.9 (n = 7)	55.5 ± 5.3 (n = 7)

Note:

To enable comparison between the three experimental conditions tested, % of I_hERG_ tail inhibition values calculated as the fractional block of outward I_hERG_ tail at −40 mV were time matched. For each drug, I_hERG_ tail was measured at steady state of block which occurred within 8–10 min of drug superfusion for dofetilide, terfenadine and thioridazine, 6‐min for ketoconazole and 3‐min for chloroquine. Columns show mean ± SEM values. The numbers in parentheses represent the number of cells tested.

* Denotes statistically significant difference from in absence of erythromycin at *p* < .05.

** Denotes significance at *p* < .01.

*** Denotes significance at *p* < .001 (One Way ANOVA with Bonferroni post‐hoc test).

### 3.4 Investigating the molecular basis for erythromycin effect on hERG pharmacological sensitivity

**Figure 3:**
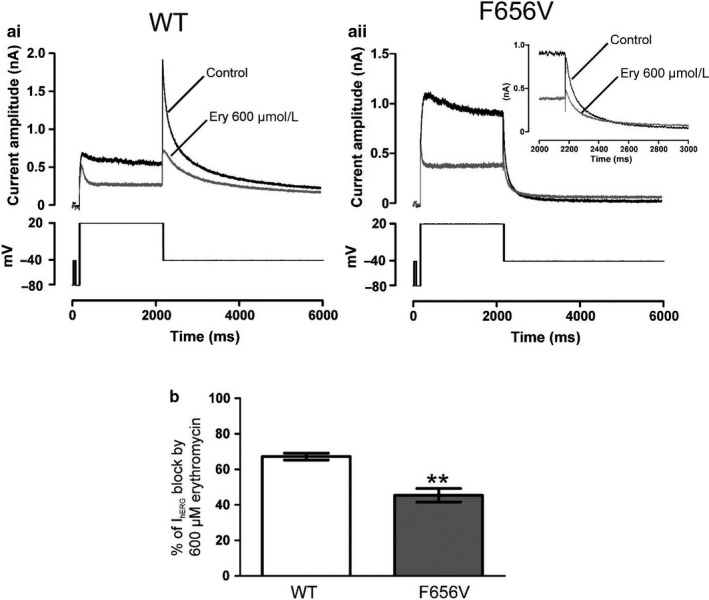
Effect of mutation at F656 on I_hERG_ block by erythromycin (a) Representative traces for WT I_hERG_ (ai) and F656V I_hERG_ (aii) before (control) and after exposure to 600 µM erythromycin (Ery) at 37ºC, using the voltage protocols in the panels below. (b) Bar charts comparing the level of block produced by 600 µM erythromycin of WT (white bar; *n* = 5) and F656V (gray bar; *n* = 5) I_hERG_ tail on repolarization to −40 mV following a 2 s depolarization from −80 mV to +20 mV. Asterisks in B denote statistical significance: ** *p* < .01 (unpaired *t* test)

It has been suggested that erythromycin may bind to an external site that would participate in the pharmacological modulation of hERG’s block by pore inhibitors (Crumb, 2014). In previous work from our laboratory mutation of the canonical inhibitor‐binding residue F656 to Ala (hERG F656A) produced only a modest reduction in erythromycin block (Duncan et al., 2006). However, differences between F656A and WT hERG channels require inward I_hERG_ measurement in high external [K^+^] to obtain sufficiently large tail currents. In contrast, the valine mutation of F656 (hERG F656V) preserves the current profile of WT hERG in normal K^+^ (Lees‐Miller et al., 2000). Therefore, we employed the F656V mutation to explore the interaction between erythromycin and the internal pore cavity where most hERG blocking drugs bind. Figure 3ai and aii, respectively, show representative traces for inhibition of WT and F656V I_hERG_ by 600 µM erythromycin, with the protocol shown in the lower panels. Mean data comparing percentage of I_hERG_ tail inhibition by 600 µM erythromycin is shown in Figure 3b. At 6 min of drug superfusion, WT and F656V I_hERG_ were reduced, respectively, by 67.2 ± 1.9% (*n* = 5) and 45.4 ± 3.8% (*n* = 5; *p* < .001 vs. WT). This modest reduction in channel sensitivity to erythromycin is in good agreement with previously reported data obtained for alanine mutation of residue F656 (Duncan et al., 2006). Upon drug superfusion, I_hERG_ tail current on repolarization to −40 mV exhibited a slowing in the rates of deactivation. Tail currents were fitted using a biexponential to derive the fast τ_1_ and slow τ_2_ time constants of deactivation in control and after 6‐min superfusion of 600 µM erythromycin. For WT I_hERG_ tails, the time constant τ_1_ was slowed: τ_1_ was 133.3 ± 22.4 ms in control and 300.9 ± 21.2 ms in erythromycin (*n* = 5; *p* < .01 vs. in control). No change to the time constant τ_2_ was observed. In contrast, both time constants of deactivation were slowed for currents recorded from the mutant channel. The time constant τ_1_ was 27.9 ± 1.5 ms in control and 64.0 ± 11.9 ms in erythromycin (*n* = 5; *p* < .05 vs. in control), whereas τ_2_ was 286.5 ± 54.2 ms in control and 665.0 ± 81.8 ms in erythromycin (*n* = 5; *p* < .05 vs. in control). This change to the time constants of deactivation resulted in a crossing‐over of F656V I_hERG_ currents as displayed in Figure 3aii and was observed in all five recordings obtained. Crossing‐over of tail currents on repolarization is typically associated with a “foot in the door” type of blockade (Ducroq, Printemps, & Le Grand, 2005; Hreiche, Plante, Drolet, Morissette, & Turgeon, 2011; Margulis & Sorota, 2008; Sánchez‐Chapula et al., 2002); it indicates that in the case of erythromycin the drug may slow closure of the deactivation gate through interaction with a noncanonical‐binding site.

**Figure 4:**
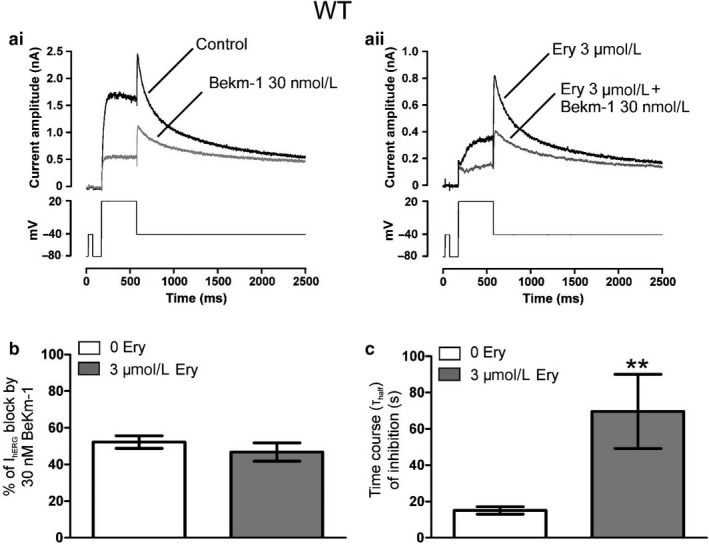
Effects of 3 µM erythromycin on I_hERG_ tail sensitivity to 30nM BeKm‐1. (a) Representative current traces for effects of 30 nM BeKm‐1 on I_hERG_ in the absence (ai) or after superfusion of 3 µM erythromycin (aii). Currents were elicited using the protocol shown in the lower panels. (b) Bar charts showing the level of block by 30 nM BeKm‐1 of WT I_hERG_ tail in the absence (white bar; *n* = 10) and presence of 3 µM erythromycin (gray bar; *n* = 6). (c) Bar charts comparing t_half_values of inhibition of WT I_hERG_ tail in the absence (white bar; *n* = 10) and presence of 3µM erythromycin (gray bar; *n* = 6). BeKm‐1 effect on I_hERG_ tail recorded on repolarization to −40mV was calculated as the mean fractional block and plotted against time to derive the value for time to half inhibition (t_half_) of I_hERG_ tail. ** denotes statistically significant difference from WT at *p* < .01 (unpaired *t‐*test)

Without a clear pattern of effects for erythromycin on hERG pharmacological sensitivity to its pore inhibitors, probing the existence of an external‐binding site by way of mutagenesis was challenging. To attempt to address this issue we used the externally acting blocker BeKm‐1, which has a known external‐binding site (Milnes, Dempsey, et al., 2003; Tseng et al., 2007), to investigate whether erythromycin interacts with, or can influence binding to, the S5‐pore linker region of the hERG channel. BeKm‐1 potency against I_hERG_ tail was assessed in the absence and in the presence of 3 µM erythromycin. Figure 4 shows the effects of 30 nM BeKm‐1 on I_hERG_ tail in the absence and following superfusion of 3 µM erythromycin; representative traces of effects of 30 nM BeKm‐1 are shown in Figure 4ai,aii with mean fractional percentages of block and values for the time to half inhibition (t_half_) of I_hERG_, respectively, shown as bar graphs in Figure 4b and 4c. Consistent with a previous study from our laboratory (Milnes, Dempsey, et al., 2003), BeKm‐1 blockade of I_hERG_ exhibited inverse dependence on duration of depolarization (data not shown). Consequently, to ensure adequate levels of I_hERG_ block, drug effects for all experiments utilizing BeKm‐1 were assessed against I_hERG_ tail elicited by a short (400 ms) depolarizing step protocol (shown in the lower panel of Figure 4ai,aii). 30 nM BeKm‐1 reduced I_hERG_ tail by 52.2 ± 3.4% (*n* = 10) (Figure 4b). In the presence of 3 µM erythromycin, I_hERG_ tail block by 30 nM BeKm‐1 was 46.8 ± 5.0% (*n* = 6; *p* > .05 vs. in the absence of 3µM erythromycin) (Figure 4b). While the effect of BeKm‐1 at steady state remained statistically unchanged, the time course of I_hERG_ tail inhibition was slowed. Figure 4c shows bar graphs for the time to half inhibition (t_half_) of I_hERG_ tail by 30 nM BeKm‐1 in the absence and in the presence of 3 µM erythromycin. In the absence of 3 µM erythromycin, evaluation of the averaged time course of I_hERG_ inhibition obtained for 10 cells yielded a t_half_ of 15.1 ± 2.1 s. In the presence of 3 µM erythromycin, t_half_ was 69.6 ± 20.4 s (*n* = 6; *p* < .01 vs. in the absence of erythromycin). These experiments showed that erythromycin was able to interfere with BeKm‐1 block of I_hERG_, suggesting that erythromycin may have an external‐binding site. However, when we tested sensitivity of F656V I_hERG_ tail to 30 nM BeKm‐1 in the absence and presence of 3 µM erythromycin, we found that erythromycin had no effect on F656V hERG block by BeKm‐1.

**Figure 5:**
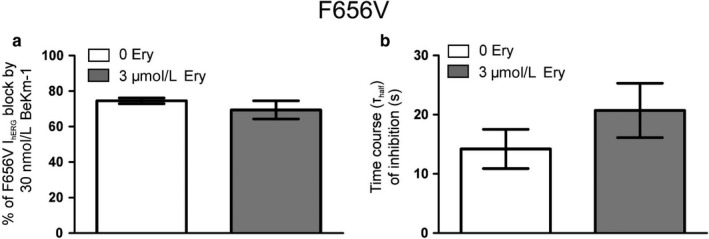
Effects of mutation F656V on erythromycin modulation of I_hERG_ sensitivity to 30 nM BeKm‐1. (a) Bar charts showing the level of block by 30 nM BeKm‐1 of F656V I_hERG_ tail in the absence (white bar; *n* = 5) and presence of 3 µM erythromycin (gray bar; *n* = 5; *p* > .05 vs. in the absence of erythromycin 3 µM [unpaired *t* test]). (b) Bar charts comparing t_half_ values of inhibition of F656V I_hERG_ tails in the absence (white bar; *n* = 5) and presence of 3 µM erythromycin (gray bar; *n* = 5; *p* > .05 vs. in the absence of erythromycin 3 µM [unpaired *t* test]). BeKm‐1 effect on I_hERG_ tail recorded on repolarization to −40mV was calculated as the mean fractional block and plotted against time to derive the value for time to half inhibition t_half_ of I_hERG_ tail. The F656V mutation increased hERG sensitivity to BeKm‐1 in the two experimental conditions tested but did not affect the rate of block (cf. Figure 4b and 4c)

Figure 5 shows bar charts for mean fractional percentages of F656V I_hERG_ block by 30 nM BeKm‐1 (Figure 5a) and corresponding mean values for T_half_ of inhibition (Figure 5b) obtained in the absence and presence of 3 µM erythromycin. 30 nM BeKm‐1 reduced F656V I_hERG_ by 74.5 ± 1.6% (*n* = 5; *p* < .001 vs. WT) with a T_half_ of I_hERG_ inhibition of 14.2 ± 3.3 s (*n* = 5; *p* > .05 vs. WT); that is, the F656V mutation enhanced BeKm‐1 block of hERG. Preapplication of 3 µM erythromycin had no effect on F656V I_hERG_ block by 30 nM BeKm‐1. In the maintained presence of 3 µM erythromycin, BeKm‐1 block of F656V I_hERG_ at steady state was 69.4 ± 5.1% (*n* = 5; *p* > .05 vs. in the absence of erythromycin) and a t_half_ of I_hERG_ inhibition of 20.7 ± 4.6 s (*n* = 5; *p* > .05 vs. in the absence of erythromycin). The enhanced sensitivity of the F656V mutant channel to 30 nM BeKm‐1 (WT and F656V I_hERG_ block by 30 nM BeKm‐1 were, respectively, of 52.2 ± 3.4% and 74.5 ± 1.6% (*p* < .001 vs. WT)) suggests an effect of the pore mutation on binding of BeKm‐1 to the external turret. Each of these observations may be encompassed within a model where the effect of the F656V mutation on the conformational properties of the external turret results in an impairment of erythromycin binding to its putative external site and an enhancement of BeKm‐1 binding to its external site. External binding of erythromycin affects (slows) the binding of BeKm‐1 and this effect is attenuated by the disruption of erythromycin binding resulting from the F656V mutation. However, an allosteric modulation by erythromycin of hERG sensitivity to BeKm‐1 through an internal‐binding site involving residue F656, cannot be ruled out.

**Figure 6:**
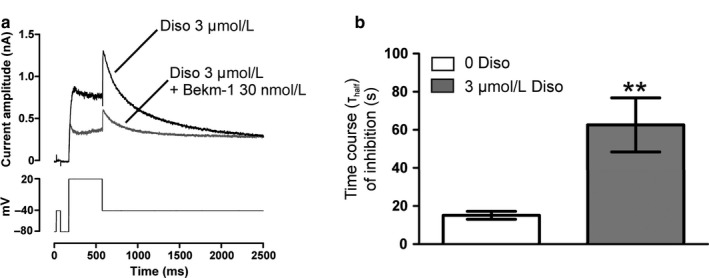
Sensitivity of I_hERG_ tail to 30 nM BeKm‐1 in the presence of 3 µM disopyramide. (a) Representative traces for I_hERG_ before and during exposure to 30 nM BeKm‐1 in the continuous presence of 3 µM disopyramide. Currents were elicited using the protocol shown in the lower panel. (b) Bar charts comparing t_half_ values of inhibition by 30 nM BeKm‐1 of WT I_hERG_ tail in the absence (white bar; *n* = 10) and presence of 3 µM disopyramide (gray bar; *n* = 7). Asterisks in B denote statistical significance: **p < .01 (unpaired *t* test)

To address this issue, we performed additional experiments with the Class Ia antiarrhythmic agent disopyramide (IC_50_ = 7–10 µM; [El Harchi et al., 2012]), which is known to access the open channel and bind within the open pore cavity at a site involving residue F656 ([El Harchi et al., 2012]; see also Methods, Drug Selection and Preparation). Figure 6 shows the effects on I_hERG_ of 30 nM BeKm‐1 in the continuous presence of 3 µM disopyramide, with representative traces of effects shown in Figure 6a and corresponding mean values for t_half_ of inhibition obtained in the absence and presence of 3 µM erythromycin shown in Figure 6b. As shown in Figure 6b, preapplication of 3 µM disopyramide delayed the time course of WT hERG block by 30 nM BeKm‐1 (*n* = 7; *p* < .01 vs. in the absence of 3 µM disopyramide). However, there was no significant effect of disopyramide on BeKm‐1 potency at steady‐state block (% of I_hERG_ block was 51.4 ± 4.6% at 6 min of drug superfusion; *n* = 7; *p* > .05 vs. in the absence of 3 µM disopyramide). These data showed that preapplication of 3 µM disopyramide delayed the time course of WT hERG inhibition by 30 nM BeKm‐1 to a similar extent to that of 3 µM erythromycin, which: (a) demonstrates communication between the distinct disopyramide (internal) and BeKm‐1 (external) binding sites; (b) by analogy, indicates that erythromycin could affect the time‐course of BeKm‐1 block without necessarily competing for a single‐binding site.

## 4 DISCUSSION

In clear contrast to an earlier study reporting a protective effect of macrolides against block of hERG overexpressed in HEK cells (Crumb, 2014), the results of this study show that pretreatment with low concentrations of erythromycin does not antagonize the inhibitory effects of normally potent hERG blocking drugs in an equivalent overexpression system. Exposure to low concentrations of erythromycin did not reduce hERG pharmacological sensitivity to the antipsychotic thioridazine and the antihistamine terfenadine. In fact, under the conditions of our study, the IC_50_ value for I_hERG_ tail inhibition by terfenadine was decreased by ~32‐fold in the presence of 3 µM erythromycin (*p* < .05 vs. no preincubation). This significant shift in sensitivity was associated with a DCI calculated value of 0.06 which indicates strong synergism (Chou, 2006). The synergetic effect was observed for the lowest concentrations of terfenadine, whereas for the highest concentrations used the effect was sub‐additive. Sensitivity to thioridazine in the presence of 3 µM erythromycin remained unchanged (*p* > .05 vs. no preincubation). Increasing the erythromycin concentration to 30 µM did not produce any further effect on either terfenadine or thioridazine potencies. Overall, the results from our investigation of the effects of low concentrations of erythromycin on recombinant channels were consistent with additive and/or synergistic effects that vary with the order of application, the concentration and nature of the pore inhibitors tested.

### 4.1 Effects of combining multiple hERG blockers: Our findings in context

Despite the fact that drug‐induced QT interval prolongation often occurs clinically with coadministration of >1 QT interval prolonging drug (Hancox et al., 2008; Yap & Camm, 2003), there are only a few in vitro studies examining the effects of the combined administration of multiple hERG blockers on the hERG channel (e.g., Crumb, 2014; Ducroq et al., 2005; Friemel & Zunkle, 2010; Hreiche et al., 2011; Kornick et al., 2003; Margulis & Sorota, 2008; Wiśniowska, Lisowski, Kulig, & Polak, 2018), and reports on the pharmacological antagonism of hERG block are scarce (Crumb, 2014; Hreiche et al., 2011). Prior to the report of a protective effect of macrolides (Crumb, 2014), one study showed that MAPD_90_‐ prolongation mediated through I_Kr_ block by the concomitant application of single concentrations of erythromycin or ketoconazole with dofetilide was less than the addition of the effects of each drug alone (Hreiche et al., 2011). This would suggest that the combination of two I_Kr_ blockers could lead to pharmacological antagonism. We did not observe any pharmacological antagonism or reversal of I_hERG_ block associated with the administration of low concentrations of erythromycin (≤30 µM) with diverse pore and nonpore hERG blockers. However, we did find, consistent with previous reports (Ducroq et al., 2005; Friemel & Zunkle, 2010; Hreiche et al., 2011; Kornick et al., 2003; Margulis & Sorota, 2008; Wiśniowska et al., 2018), that the paired combination of two hERG inhibitors is associated either with independent or additive/synergistic effects. In our hands, pretreatment with erythromycin was associated with either strong synergism (terfenadine, chloroquine), slightly less than additive effects (dofetilide and thioridazine) or limited effects (ketoconazole and BeKm‐1). When erythromycin was applied after steady‐state hERG channel block by thioridazine, dofetilide or chloroquine, erythromycin superfusion was associated with a potentiation of hERG block suggesting independent actions. In contrast, currents preinhibited by terfenadine or ketoconazole were insensitive to erythromycin. The variability in the nature and degree of the allosteric interactions seen in our paired combination studies suggests that whether drugs had similar‐ or dissimilar‐binding sites within the channel's pore cavity does not greatly influence the outcome of combining blockers. However, most studies examining the effects of combining two hERG inhibitors reported that drugs binding within the pore cavity of the hERG channel are most likely to interact with drugs that do not (e.g., Ducroq et al., 2005; Friemel & Zunkle, 2010; Hreiche et al., 2011; Kornick et al., 2003; Margulis & Sorota, 2008; Wiśniowska et al., 2018). As an example, Friemel and Zunckler's study characterized the potential interactions between a drug inhibitor binding within the pore cavity with drugs binding elsewhere on the hERG channel (Friemel & Zunkle, 2010); drugs were selected on the basis of their specific interaction with the hERG channel. They showed that compounds with overlapping‐binding sites within the pore cavity do not interact and their pairwise combination was associated with additive inhibitory effects (terfenadine in combination with dofetilide). In contrast, the externally acting hERG inhibitor CnErg1 slightly antagonized the inhibitory effects of terfenadine, whereas chlorobutanol, a drug thought to bind to an unidentified site outside the pore cavity, was found in combination with terfenadine to inhibit I_hERG_ synergistically. Surprisingly, fluvoxamine, which like erythromycin was shown to have limited interaction with residue F656 and has been suggested to bind at the outer mouth of the pore (Milnes, Crociani, et al., 2003; Mitcheson, 2003), did not influence I_hERG_ block by terfenadine. A subsequent modeling study, however, showed that although fluvoxamine has weak interactions with residue F656, its trifluoromethyl group could interact with the nonaromatic residues T623 and S624 (Stansfeld et al., 2007). This suggests that terfenadine and fluvoxamine may have overlapping‐binding sites and thus explain the lack of interactions when the two drugs are simultaneously applied (Friemel & Zunkle, 2010). These findings highlight the complexity of the interactions of drug compounds with their respective binding sites and underpin the need for a comprehensive approach combining both functional studies with evidence for the location of binding sites. The data presented in this report are consistent with an external site for erythromycin, but we cannot, however, rule out some contribution from erythromycin binding to an intracellular domain of hERG. Erythromycin may therefore act through two different binding sites, one intracellular site involving residue F656 likely to be involved in low affinity block of the channel (Duncan et al., 2006), and another site located on the extracellular side of the channel that may participate in the modulation of hERG pharmacological block by pore inhibitors. Our data also revealed possible allosteric interactions between binding sites on the external and internal face of the channel, which mirrors previous evidence for communication between the extracellular turret of hERG and the drug‐binding site below the selectivity filter, especially in relation to inactivation gating (Ju et al., 2009; Perrin, Kuchel, Campbell, & Vandenberg, 2008). The evidence for internal and external sites for erythromycin makes it challenging, however, to identify a clear pattern of effects for the concomitant application of erythromycin with multiple hERG blockers.

### 4.2 Implications and limitations

The present work and the previous study by Crumb (Crumb, 2014) each highlight the potential for distinct drug‐binding sites on hERG that may interact allosterically. However, despite broadly similar experimental conditions, including the use of a similar heterologous expression system for stable expression of WT hERG, similar recording solutions near physiological temperatures and equivalent incubation times with low concentrations of erythromycin; they differ markedly in respect of the presence/absence of a protective effect of erythromycin on I_hERG_ inhibition by other drugs. Experimental details regarding how pretreatment with a low erythromycin concentration was performed in (Crumb, 2014) are sparse and this precludes detailed comparison of the two investigations in this regard. The basic voltage protocol used to elicit I_hERG_ in the two studies has some differences and it is conceivable that these might have contributed to the differences in results of the two studies. However, the differences in holding potential of −75 mV in (Crumb, 2014) versus −80 mV here and test voltage (+10 mV in (Crumb, 2014) and +20 mV here) are small and not anticipated to affect fundamentally the results obtained. A depolarizing test pulse duration of 2 s was used here versus 500 ms in the earlier study (Crumb, 2014). At near physiological temperatures hERG should be substantially activated at the test potentials used within 500 ms (Zhou et al., 1998). The longer pulse duration used here might allow longer for drugs to bind to gated channels during membrane depolarization. However, steady‐state block for high‐affinity drugs that become trapped in the hERG channel (such as terfenadine: Stork et al., 2007) may not be much impacted by this difference. Indeed, we performed some additional experiments with thioridazine to compare effects of 500 ms depolarizing steps to +20mV and 2s depolarizing steps on drug effects on I_hERG_ tails at −40mV. We found 3 µM erythromycin alone to inhibit I_hERG_ tail by 24.8 ± 3.9% with a 500 ms test pulse (*n* = 6; *p* > .05 vs. a 2s depolarizing step). 100 nM thioridazine reduced WT I_hERG_ tail by 52.0 ± 4.4% with a 500 ms test pulse (*n* = 5; *p* > .05 vs. a 2s depolarizing step) and 65.1 ± 4.1% (*n* = 5; *p* > .05 vs. in the absence of 3 µM erythromycin and *p* > .05 vs. a 2s depolarizing step), respectively, in the absence and presence of 3 µM erythromycin. These results argue against a major role for voltage protocol in underlying the different findings of the two studies. The basis for the differences in findings of the two studies is therefore not clear at the present time.

The ultimate aim of a comprehensive understanding of I_Kr_/hERG pharmacology in relation to diLQTS is enhanced safety of drug use in patients and in drug development (Hancox et al., 2008; Sanguinetti & Tristani‐Firouzi, 2006). Among the known risk factors increasing the susceptibility of individuals to diLQTS, the coadministration of more than one hERG‐blocking drug (Hancox et al., 2008; Yap & Camm, 2003) is a common practice in a clinical setting and an issue that physicians have to take into account when coprescribing drugs. Drug induced QT prolongation resulting from the combination of two or more inhibitors that share metabolic pathway(s) (this can raise plasma drug levels) are well documented (Hancox et al., 2008; Vieweg & Wood, 2004) and coadministration of erythromycin with other drugs that inhibit or are metabolized by CYP3A4 or with QTc prolonging drugs has been identified as a major risk factor (reviewed in Hancox et al., 2014). To our knowledge, this study is the first to characterize in vitro interactions of erythromycin with a range of internal and external hERG blockers. Our report is in line with recent recommendations from the European Medicines Agency (EMA) advising pharmacodynamics disruption (PD) interaction studies when two drugs, likely to be used concomitantly, compete for the same target (http://www.ema.europa.eu/docs/enGB/documentlibrary/Annualreport/2016/05/WC500206482.pdf). Our study provides further understanding of hERG blocking drug interactions, in particular involving erythromycin, and could potentially contribute to better preclinical assessment of the arrhythmogenic risk related to polypharmacy. It also suggests that caution should be exercised in considering administration of more than one hERG interacting drug to mitigate drug‐induced LQTS, as the preponderance of data suggest that protection is unlikely to occur.

This study scrutinized the effects of erythromycin modulation of hERG sensitivity to a series of pore (internal) and external inhibitors at the channel level. It therefore excluded any interference from metabolic pathways mediated by the enzymes of the cytochrome P450 system known to metabolize erythromycin extensively. Erythromycin potency against isoenzymes of the cytochrome P450 is well documented to cause increased serum concentration of terfenadine, and thereby enhanced sensitivity to drug‐induced QT interval prolongation (Biglin, Faraon, Constance, & Lieh‐Lai, 1994; Paris, Parente, Bruschetta, Guzman, & Niarchos, 1994). The synergistic interaction resulting from the combination of terfenadine with erythromycin as described here could constitute an additional factor to explain the increased susceptibility to QT prolongation consecutive to the coadministration of both inhibitors.

## 5 CONCLUSIONS

Our data indicate that in the setting of drug‐induced delayed cardiac repolarization and associated arrhythmia, reversing hERG channel blockade by way of pharmacological antagonism with low concentrations of the macrolide antibiotic drug erythromycin would not constitute an attractive adjunct or replacement treatment. Erythromycin appeared to potentially act through one intracellular‐binding site and one extracellular one that may participate in the modulation of hERG pharmacological block by some of hERG’s pore inhibitors. Utilizing alanine scanning mutagenesis, interactions of the S5‐pore linker with the intracellular regions of the channel involved in hERG block could be probed in order to understand allosteric interactions between external and internal (pore) drug sites in hERG.

## CONFLICT OF INTEREST

None.
